# Childhood Trauma and Aggression: The Role of Epistemic Trust

**DOI:** 10.1007/s40653-026-00841-2

**Published:** 2026-03-17

**Authors:** Carlo Garofalo, Veronica Raspa, Elisa Delvecchio, Irene Aiolfi, Daniela Laricchiuta, Richard Howard, Claudia Mazzeschi

**Affiliations:** 1 Department of Philosophy, Social Sciences and Education, University of Perugia, Perugia, Italy; 2 Department of Humanistic Research and Innovation, University of Bari Aldo Moro, Bari, Italy; 3 Institute of Mental Health, University of Nottingham, Nottingham, United Kingdom

**Keywords:** Child maltreatment, Cycle of violence, Aggressive behavior, Violence, Mentalization, Attachment

## Abstract

The relation between childhood trauma and aggression is well-established. Yet, empirical findings are inconclusive regarding specific associations between types of childhood trauma and distinct types of aggression, as well as regarding possible mechanisms explaining their association. We examined associations that physical and emotional abuse, physical and emotional neglect, and sexual abuse had with four aggression types according to the Quadripartite Violence Typology, as well as their indirect effects through epistemic trust, mistrust, and credulity in a community sample of 251 adult participants (54.2% women; *M*_*age*_ = 31.33, *SD* = 11.20). Levels of childhood trauma were positively linked to higher levels of aggression and lower levels of epistemic trust. At the subscale-level, emotional abuse had the most consistent associations. Epistemic mistrust was consistently linked to aggression and helped explain relationships between childhood traumatic experiences and aggression. Accounting for the overlap across types of childhood trauma, only sexual abuse exerted unique effects, while most of the main findings were accounted by the shared variance among physical and emotional abuse and neglect. Childhood trauma emerged as a consistent yet non-specific risk factor for different types of aggression. Mistrust in others as sources of trustworthy information may be one pathway linking early traumatic experiences with later aggressive behavior.

**Supplementary Information:**

The online version contains supplementary material available at https://doi.org/10.1007/s40653-026-00841-2.

## Introduction

Childhood maltreatment is a widespread public health problem affecting millions of children worldwide (World Health Organization; WHO, 2006). The WHO defines child maltreatment as “all forms of physical and/or emotional ill-treatment, sexual abuse, neglect or exploitation, resulting in actual or potential harm to the child’s health, survival, development or dignity” (Bernard et al., 2017, p. 15). It is estimated that at least 25% of individuals worldwide have experienced some form of maltreatment during childhood (WHO, 2006). Not only does this represent a violation of children’s rights, but it also has profound and long-lasting consequences for their mental health, giving rise to the need for more in-depth research to guide effective interventions in the aftermath of early traumatic experiences (Humphreys et al., 2020; Norman et al., 2012; Perry, 2004; WHO, 2006).

Although traumatic experiences are broader in scope than child maltreatment, for the purpose of the present study we use the two terms interchangeably with the understanding that our use of the word “trauma” refers to experiences of child maltreatment as operationalized in the widely used Childhood Trauma Questionnaire (see Method section). Repeated exposure to traumatic experiences is more damaging than isolated incidents of stress, making it important to study the severity of exposure to early traumatic experience beyond its (mere) occurrence. In the most extreme cases, the psychological damage can be profound, chronic, and difficult to treat, even later in life (Perry, 2004). Childhood maltreatment has been associated with a wide range of psychopathology consequent upon the child’s traumatic experiences, as well as with aggressive and violent behavior (Leenarts et al., 2013). Systematic reviews and meta-analyses (e.g., Baldwin et al., 2023), have found consistent associations between childhood maltreatment and psychiatric disorders, and various studies have corroborated links between childhood maltreatment and aggression in adulthood (Caspi et al., 2014; Norman et al., 2012).

Among the many forms of child maltreatment, sexual abuse has often been studied in isolation (e.g., Liu, 2017), but different types of maltreatment often occur simultaneously (Petersen et al., 2014). For this reason, it is important to examine how different types of childhood maltreatment are related to maladaptive outcomes. In fact, early experiences of maltreatment (such as physical or emotional abuse and neglect, and sexual abuse) may have shared and unique effects on the onset of psychopathology compared to one another (Humphreys & Zeanah, 2015). As such, differential effects may also concern relationships between different forms of child maltreatment and distinct forms of aggression.

### The Quadripartite Violence Typology in the Cycle of Violence

In recent decades, prospective and longitudinal studies have begun to emerge, supporting the hypothesis of the so-called “cycle of violence”, which suggests that maltreated children are at increased risk of developing aggressive or otherwise antisocial behavior that persists into adulthood (Ehrensaft et al., 2003; Fitton et al., 2020; Maxfield & Widom, 1996; Ogloff et al., 2012; Schuck & Widom, 2021; Widom, 1989; Widom, 2017). In particular, this hypothesis suggests that physically abused children are at increased risk of acting violently as adults (Maxfield & Widom, 1996). In examining the long-term effects of abuse and maltreatment on rates of delinquency and violence, Maxfield and Widom (1996) found that maltreated children were significantly more likely to be arrested for both young and adult crimes than non-maltreated children. This stream of research has provided consistent evidence of links between early traumatic experiences and aggression (Caspi et al., 2014; DeLisi et al., 2018; Leenarts et al., 2013; Norman et al., 2012). However, some gaps remain open; specifically: (1) whether different forms of maltreatment have distinct associations with aggression; (2) whether different forms of aggression are associated with early maltreatment in different ways; and (3) what mechanisms may explain these associations.

**Fig. 1:**
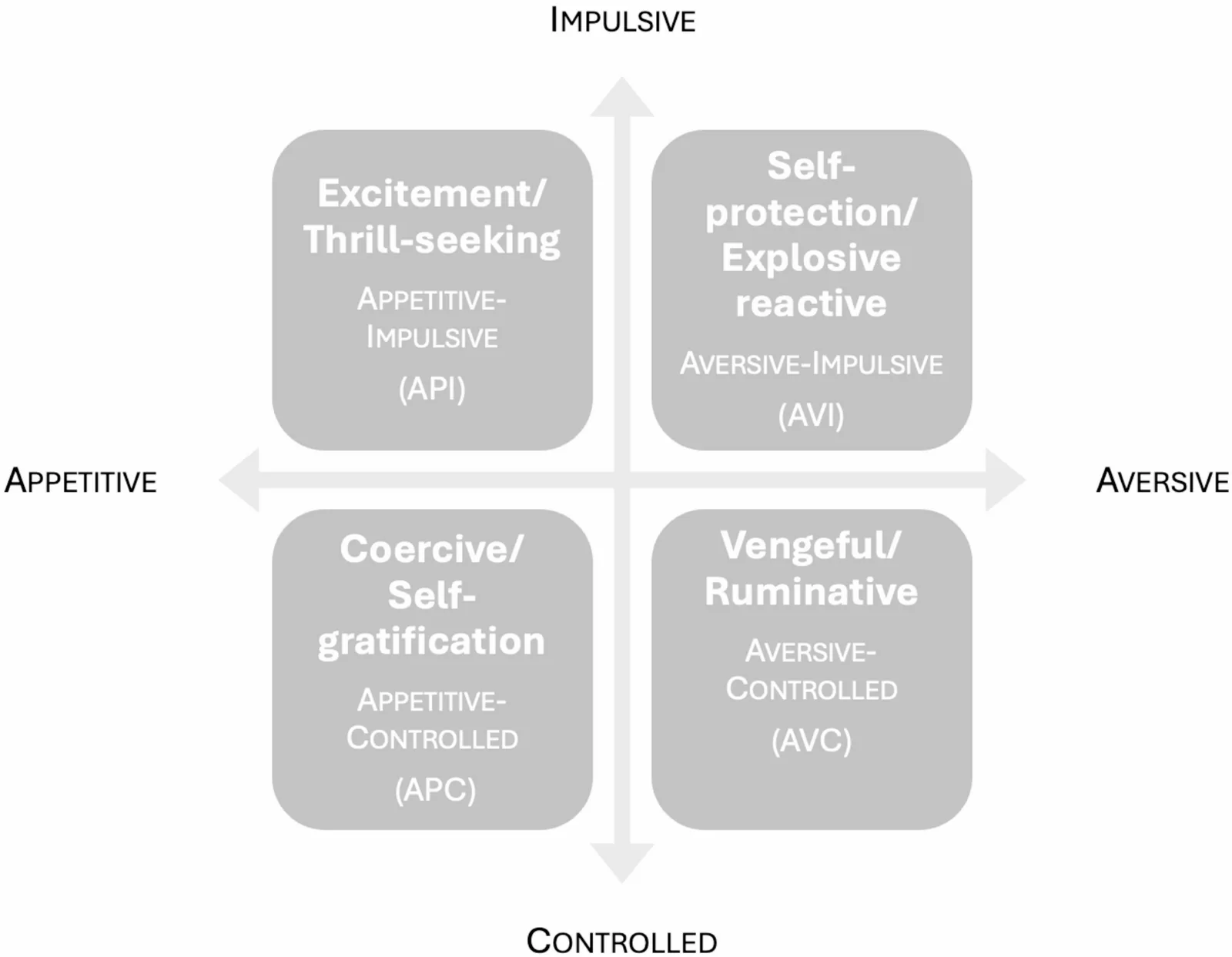
A graphical depiction of the quadripartite violence typology

To add nuance to the study of the associations between early traumatic experiences and aggression, it may be useful to consider the “Quadripartite Violence Typology” (QVT), developed to capture the motivational heterogeneity of violence (Howard, 2009; Howard et al., 2009). QVT extends and revises the dichotomy between instrumental and affective aggression, by intersecting two fundamental dimensions of aggression giving rise to four distinct forms of aggressive behavior (see Fig. 1). Specifically, on one dimension it conceptualizes aggression as motivated either by an aversive response (defense from a threat) or from an appetitive motivation (predatory act to obtain an emotional or material reward). The second dimension concerns the degree of self-control exerted in the aggressive behavior, which could be either impulsive or controlled. The intersection of these two dynamic dimensions can give rise to aggression that is aversively motivated and impulsive (self-protection/explosive reactive), aversively motivated and controlled (vengeful/ruminative), appetitively motivated and impulsive (excitement/thrill-seeking), or appetitively motivated and controlled (coercive/self-gratification). Although there is evidence the early traumatic experiences are linked to both reactive and proactive aggression, with stronger effects for the former than the latter (Eisenbarth & Garofalo, 2021; McRae et al., 2022), to our knowledge no studies have investigated associations among different forms of early traumatic experiences and the four types of aggression conceptualized in the QVT, let alone possible mechanisms explaining these associations.

### Trauma and Aggression: The Role of Epistemic Trust

The relationship between early traumatic experiences and aggressive behavior is complex and multifaceted, and likely explained by several potential factors partly or fully accounting for their relationship. To examine factors that account for a relationship between two constructs, researchers typically employ statistical mediation analysis. However, mediation analysis imply a temporal ordering among the study variables which require longitudinal data to assess for prospective associations. In contrast, cross-sectional mediation analysis that aim to examined whether a significant amount of the variance shared by two variables could be accounted for by a “mediating” variable have been referred to as a-temporal mediation (Winer et al., 2016) or associational variable analysis (Weems, 2025). Throughout this study, we refrain from using the term mediation when based on cross-sectional data, and instead use terms like “indirect effect”, “explains associations” or “accounts for the association” when referring to variables that are modeled as cross-sectional mediator so as to not imply untested temporal relationships among variables.

Among putative variables explaining the association between childhood traumatic experiences and externalizing behaviors, prominent examples are individual differences in emotional reactivity and sensitivity to rewards and punishments (Weeland et al., 2015). More specifically, several psychological mechanisms have been proposed to explain how early traumatic experiences may contribute to aggressive behaviors. These psychological mechanisms include, but are not limited to: emotional regulation, impulsivity and disinhibition, psychopathic traits, attachment, mentalizing, cognitive processing, identity and self-esteem (Dodge et al., 1995; Dutton, 2007; Eisenbarth & Garofalo, 2021; Kim & Cicchetti, 2006; Malcorps et al., 2024; Meddeb et al., 2023; Schwarzer et al., 2021; Shields & Cicchetti, 1998; Stagaki et al., 2022; Taubner et al., 2016; Tironi et al., 2024). A recent review more specifically focused on the interpersonal transmission of violence perpetration has identified five underlying mechanisms in prospective studies: normalizing attitudes towards violence and harmful gender norms; modeling and imitation; poor emotion regulation; high socio-economic vulnerability; and impairments in interpersonal relationships (Meinck et al., 2025). Of these, emotion dysregulation and impaired relationships emerged as the most important mechanisms linking violence *victimization* and violence *perpetration*, a topic germane to the focus of the present investigation.

Here, we propose that a relatively new concept – epistemic trust – may serve as another mechanism that accounts for the relationship childhood trauma and aggression serving as an “associational variable”. Epistemic trust is the ability to consider information from others as relevant and applicable to one’s own experience, thus facilitating learning and social adaptation (Fonagy et al., 2017). Epistemic trust is not an innate ability but depends largely on the family and social environment in which a person grows up. It refers to an individual’s ability to see information provided by other people as relevant to one’s well-being, meaningful to one’s experience, and applicable across different situations (Campbell et al., 2021). Further, it has been hypothesized that epistemic trust is a key element in protecting against the development and persistence of psychological disorders (Fonagy et al., 2015; Luyten et al., 2020). However, further systematic research is needed to explore the possible role of epistemic trust in explaining links between early traumatic experiences and aggression, which is plausible based on theoretical grounds and indirect empirical evidence described in the following paragraphs, adopting attachment theory as a cohesive theoretical framework. By relying on cross-sectional data, we refer to a potential role of epistemic trust as it could be supported by findings that epistemic trust helps account for (part of) the relationship between childhood trauma and aggression.

Attachment and epistemic trust are two closely interconnected, but distinct processes in child development (Fonagy & Allison, 2014). In fact, attachment facilitates the reliable transmission of knowledge, lowering epistemic vigilance thanks to appropriate ostensible signals from the caregiver, fostering intergenerational transmission of secure attachment and mentalizing (Fonagy & Allison, 2014). Csibra and Gergely’s (2009) natural pedagogy theory proposed that infants learn from caregivers through ostensive signals (e.g., eye contact, affectionate tone), which reduce epistemic vigilance and foster trust. This process enhances secure attachment and facilitates the transmission of culturally relevant knowledge. In relational contexts of development characterized by a secure attachment, epistemic trust can develop in a healthy way (Fonagy & Allison, 2014). Growing up, the infant must overcome natural epistemic vigilance to learn cultural information essential for survival (Fonagy & Allison, 2023).

If secure attachment promotes trust in oneself and in the caregiver, insecure or disorganized attachment styles contribute to excessive mistrust, and this may occur in the context of attachment relationships characterized by child maltreatment (Schmitz et al., 2023). As such, epistemic trust represents a fundamental aspect in the development of interpersonal relationships, since it allows useful knowledge to be accepted and internalized. However, insecure environments such as those characterized by childhood traumas can impair the development of this capacity, generating maladaptive forms of epistemic trust (Schmitz et al., 2023; Sprecher et al., 2022; Sperber et al., 2010). Maladaptive epistemic trust can be manifested in three main forms. First, *epistemic mistrust* leads to extreme suspicion regarding other people’s information, resulting in the individual rejecting or ignoring what is communicated to him/her. Second, *epistemic credulity* translates into an excessive readiness to believe things and a vulnerability to manipulation and deception. Third, *epistemic vigilance* involves an inconsistent and unstable attitude towards information sources. These dysfunctional forms of epistemic trust can, in turn, lead to rejection of social information, thereby interfering with the establishment of positive social relationships and increasing the risk of developing maladaptive behaviors such as aggression (Badoud et al., 2015; Suchman et al., 2018; Talia et al., 2021).

Individuals with dysfunctional epistemic trust may also tend to expect that others will not believe them, leading to a perception of aggression as an effective strategy to receive attention and induce compliance from the listener (Talia et al., 2021). While it makes conceptual sense that exposure to early traumatic experiences interferes with the development of epistemic trust, few studies have directly explored the hypothesis of a link between epistemic trust and aggression, and whether epistemic trust can function as an associational variable in the child maltreatment-aggression link. Research on the role of epistemic trust as a potential factor explaining associations between childhood traumatic experiences and adult aggression has so far provided only indirect, yet significant, evidence. For instance, children with disorganized attachment often use controlling and aggressive means of communication after brief separations from their parents (Main & Cassidy, 1988). In adults, found that distrust accounted for the relation between insecure attachment and personality disorders in people who offended sexually against minors. LaMotte and colleagues (2016) showed that general distrust accounted for the link between exposure to traumatic events and occurrences of domestic violence in adult couples. Further, Locati et al. (2023) reported that distrust toward parental figures accounted for the relationship between mentalization abilities and externalizing symptoms in adolescents. Finally, a recent scoping review (Chokhani et al., 2025) synthesized evidence emerging from 15 studies supporting the role of trust problems (including epistemic trust) in the link between interpersonal childhood adversity and internalizing, externalizing and personality disorder symptoms.

### The Present Study

Considering the above background and to fill gaps in the extant literature, the present study examined associations among different forms of childhood trauma, epistemic trust, and aggression. Overall, the main aim of the study was to examine whether childhood traumatic experiences would be associated with aggression, and whether epistemic trust would help explain this association. More specifically, we expected that childhood traumatic experiences would be associated to all four forms of aggression postulated by QVT, with relatively stronger effects for aversively driven than for appetitively driven forms. Next, we expected that epistemic trust would be negatively related to childhood traumatic experiences and aggression, while epistemic mistrust and credulity would be positively associated with childhood traumatic experiences and aggression. Further, we tentatively expected epistemic trust (negatively), epistemic mistrust and credulity (positively) to account for associations between childhood traumatic experiences and aggression. Regarding dimensions of epistemic trust, we did not have a-priori hypotheses regarding specific forms of aggression, although it makes intuitive sense to expect that dysfunctions in epistemic trust may be especially relevant for aversive forms of aggression, characterized by perceived threat or insult received from others. Finally, in an exploratory fashion, we examined the same associations also focusing on distinct forms of childhood traumatic experiences (i.e., physical abuse and neglect, emotional abuse and neglect, and sexual abuse) to examine whether any of those associations would be accounted for by unique *versus* shared components of the different types of childhood traumatic experiences.

## Methods

### Procedure and Participants

A total of 264 adults were recruited from the Italian general population. A total of 16 master’s level psychology students recruited participants as part of their thesis or traineeship projects. They were instructed to recruit participants using snowball sampling and expanding recruitment beyond their direct contacts so that participants were recruited from the broadest possible range of socio-demographic characteristics. Hence, recruitment was conducted on a person-by-person basis, and once personal contact was established, a link was sent to complete the survey online. To detect careless responding, we used three items drawn from a validated infrequency validity scale (Christian & Sellbom, 2016). These items were structured as statements that were either highly improbable or impossible, making it unlikely for respondents to endorse them if they were sincerely engaging with the survey. The following items were strategically distributed across different sections of the survey: “I am close personal friends with the prime minister of Zanzibar”, “I am allergic to water”, and “I wrote three best-selling novels last year”. For scoring purposes, any instance where a participant endorsed one of these statements was recorded as an infrequency hit, and participants who endorsed more than one of those items were excluded from further analyses. This led to the exclusion of 13 participants (4.9%). Hence, the final sample included 251 participants (54.2% women; 45% men; 0.8% preferred not to answer), with a mean age of 31.33 years (*SD* = 11.20; range: 18–68). All participants were Italian citizens, although 6 individuals were born outside of Italy. Of those 6, only one disclosed the country of origin (Dominican Republic) and all had been living in Italy for 12 years or longer (specifically for: 12, 17, 18, 30, 33, 41 years). The participants’ educational backgrounds varied. The majority held a bachelor’s degree (31.1%), while 4% had completed lower secondary education, 4.4% had a vocational high school diploma, 23.9% had upper secondary education, and 10% had completed post-diploma training or attended at least one year of university. Additionally, 22.3% held a master’s degree, 3.6% completed a post-master’s specialization course, and 0.8% had earned a PhD. Regarding marital status, 39% of participants were in a relationship, 32.3% were single, 23.9% were married or living with their partner, 3.2% were separated or divorced, and 1.6% did not fit into the provided categories. In terms of occupation, 37.1% were students, 25.1% were employees, 9.6% were manual workers, 8.8% were self-employed, 4% were unemployed, 3.2% were entrepreneurs, and 1.2% were managers. One participant was retired, and 10.8% did not fit into any of the provided categories. Regarding the total net income earned by participants’ families, the responses varied: 19.1% earned more than €3000, 16.3% earned between €2501 and €3000, 15.1% earned between €1001 and €1500, 13.5% earned between €2001 and €2500, 13.1% earned between €1501 and €2000, and 3.2% earned below €1000. Finally, 19.5% preferred not to answer. Explicit written informed consent was obtained from all participants. No incentives for participation were provided, and the study was approved by the Ethics Committee of [masked for the purpose of peer-review].

### Measures

#### The Childhood Trauma Questionnaire—Short Form (CTQ-SF; Bernstein et al., 2003; Sacchi et al., 2018)

The CTQ-SF is a 28-item questionnaire that assesses the presence and severity of five types of childhood traumatic experiences of abuse and neglect during childhood: emotional abuse (EA), sexual abuse (SA), physical abuse (PA), emotional neglect (EN), and physical neglect (PN). Responses are rated on a 5-point Likert scale reflecting the frequency of maltreatment episodes (0 = never, 4 = very often). High scores are correlated with depressive, dissociative, and post-traumatic stress disorder symptoms (Grassi-Oliveiraz & Stein, 2008).

The CTQ-SF uses specific clinical cutoff values, varying by subscale, to distinguish individuals with minimal or no trauma exposure from those reporting childhood maltreatment. Since this study’s sample was non-clinical, we used the none-mild range as the threshold for childhood trauma exposure. The cutoff scores, derived from Bernstein et al. (1998), are as follows: EA ≥ 9, PA ≥ 8, SA ≥ 6, EN ≥ 10, PN ≥ 8.

The Italian version of the questionnaire demonstrated good reliability and validity, with Cronbach’s alpha values ranging from 0.88 to 0.96 for the sample of young Italian female adults (Sacchi et al., 2018). In the present study, Cronbach’s alpha values for Emotional Abuse was 0.82; for Physical Abuse 0.81; for Sexual Abuse 0.92; for Emotional Neglect 0.87; and for Physical Neglect 0.68. the total CTQ scale presented a Cronbach’s alpha of 0.90.

#### The Epistemic Trust Mistrust Credulity Questionnaire (ETMCQ; Campbell et al., 2021; Liotti et al., 2023)

The ETMCQ is a 15-item questionnaire that assesses three dimensions: Trust, Mistrust, and Credulity. Responses are measured on a 7-point Likert scale, with options ranging from “strongly disagree” (= 1) to “strongly agree” (= 7), with the option “neither agree nor disagree” in the middle (= 4). The questionnaire showed good reliability and validity in an Italian study conducted on adults (Liotti et al., 2023), with Cronbach’s alpha values ranging from 0.67 to 0.72. In the present study, Cronbach’s alpha values for each dimension were found to be: α = 0.67 for Trust, α = 0.60 for Mistrust and α = 0.70 for Credulity. The total ETMCQ scale showed an α of 0.71.

#### The Angry Aggression Scale (AAS; Bjørnebekk & Howard, 2012)

The AAS is a 20-item self-report questionnaire that measures four types of angry aggression: self-protection/explosive reactive (aversive impulsive; AVI), vengeful/ruminative (aversive controlled; AVC), excitement/thrill seeking (appetitive impulsive; API), and coercive/self-gratification (appetitive controlled; APC): see Fig. 1. Each item was answered on a 4-point scale with the following options: 4 = very true for me, 3 = somewhat true for me, 2 = somewhat untrue for me, and 1 = not true for me at all. The instrument showed good psychometric characteristics in the Norwegian validation study with adolescents in Norway, Cronbach’s alphas for the subscales were (Cronbach’s alphas for AAS were: 0.91 for excitement/thrill seeking (API), 0.85 for coercive/self-gratification (APC), 0.86 for vengeful/ruminative (AVC), and 0.82 for self-protection/explosive reactive (AVI); Bjørnebekk & Howard, 2012), as well as in a Dutch adult community sample (Bant et al., 2025). The AAS was translated into Italian for the purpose of the present study following a standard forward-and-back-translation procedure in consultation with the author of the original measure (Richard Howard). In the present study the Alpha values were 0.76 for API, 0.65 for APC, 0.71 for AVI, 0.80 for AVC, and 0.86 for the whole AAS scale.

### Data Analysis

**Fig. 2:**
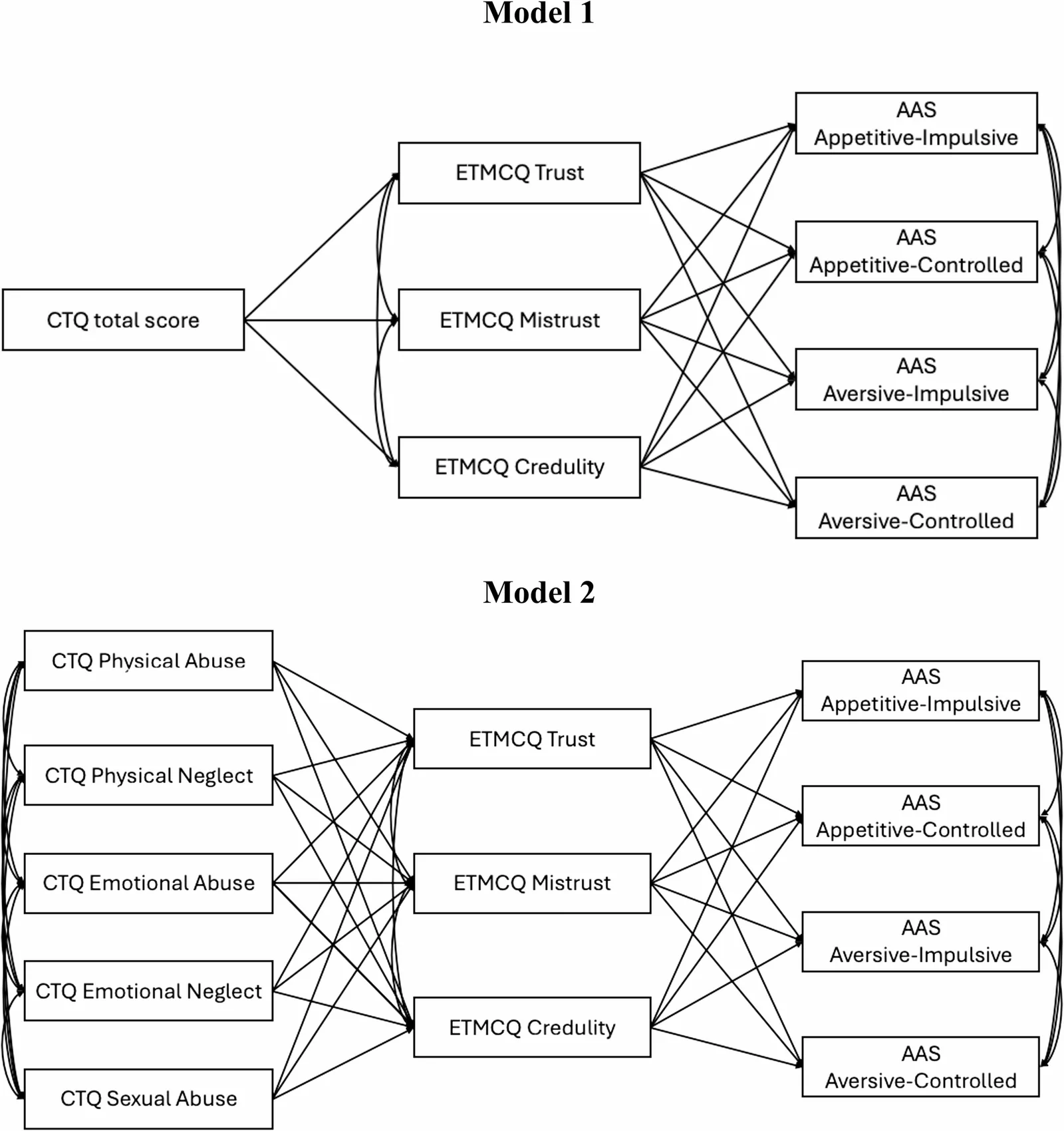
Graphical depiction of the path analysis models. Note. CTQ = total score for Childhood Trauma Questionnaire. ETMCQ = Epistemic Trust Mistrust and Credulity Questionnaire. AAS = Angry Aggression Scale. Path coefficients for Model 1 are reported in Table 2. Path coefficients for Model 2 are reported in Table 3 (only significant indirect effects) and Supplementary Materials (full model details). Residual variances for all variables were specified in the model but are not depicted for ease of reading

Data analysis was performed using IBM SPSS Statistics 27.0 software and MPlus version 8 (IBM Corp., 2020; Muthén & Muthén, 1998–2017). Because the survey was administered online with forced response options, no missing data were present in the dataset. No major violations of the assumptions required for the data analytic approach used were noted. Descriptive statistics were first calculated, followed by Pearson correlation analyses to examine the relationships among childhood traumatic experiences (total and subscale scores), epistemic trust dimensions, and aggressive behavior types. Next, mediation analyses (or associational variable analyses; Weems, 2025) were performed in Mplus using path analysis within a structural equation modeling framework with observed variables only, in a fully saturated model, to explore the role of epistemic trust, mistrust and credulity in accounting for the relationships between the traumatic experiences in childhood and the various types of aggressive behavior. We elected for a fully saturated model that also specified relationships among the ETMCQ and among the AAS subscales because (1) research has been inconsistent regarding associations across ETMCQ scales and, even in those cases where the correlations were not statistically significant, the magnitude of the associations was not trivial; and (2) AAS subscales consistently show moderate inter-correlations. Path analyses were conducted first using the CTQ-SF total score, and then with the five CTQ-SF subscales entered simultaneously as predictors. Similarly, in all path analyses, the three ETMCQ subscales were entered simultaneously as parallel mediators, and the four AAS subscales simultaneously as outcomes. The significance of indirect effects was assessed via bootstrapping with 5,000 resamples, and 95% bias-corrected confidence intervals (CIs) were computed. Indirect effects were considered statistically significant if the CIs did not include zero. A graphical depiction of the path analysis models is shown in Fig. 2.

## Results

### Descriptive and Correlational Analyses

Descriptive statistics and bivariate correlations for all study variables are reported in Table 1. Within measures, the CTQ-SF subscales were all positively related to one another with moderate to strong effects, except Sexual Abuse which had weaker and less consistent associations with other subscales. Based on the cutoffs for at least mild levels of trauma as measured by the CTQ-SF, 32.7% of the sample scored above the cutoff for Emotional Abuse, 13.1% for Physical Abuse, 6.4% for Sexual Abuse, 43.4% for Emotional Neglect, and 11.6% for Physical Neglect. The ETMCQ Trust scale was unrelated to Mistrust and positively related to Credulity, with small effect size. Mistrust and Credulity were positively related with a moderate effect size.

**Table 1:** Descriptive statistics and correlations for study variables

Variable	M	SD	^1^	^2^	^3^	^4^	^5^	^6^	^7^	^8^	^9^	^10^	^11^	^12^	^13^
1. API	1.08	0.25	*–*												
2. APC	1.13	0.26	0.38^**^	*–*											
3. AVI	1.43	0.45	0.39^**^	0.49^**^	*–*										
4. AVC	1.78	0.59	0.33^**^	0.48^**^	0.58^**^	*–*									
5. TRUST	5.01	1.07	− 0.15^*^	0.03	0.06	0.08	*–*								
6. MISTRUST	3.82	1.07	0.20^**^	0.27^**^	0.27^**^	0.22^**^	0.00	*–*							
7. CREDULITY	2.75	1.14	0.02	0.14^*^	0.17^**^	0.11	0.19^**^	0.42^**^	*–*						
8. CTQ_EA	7.94	3.61	0.24^**^	0.21^**^	0.28^**^	0.29^**^	− 0.05	0.23^**^	0.18^**^	*–*					
9. CTQ_PA	6.03	2.19	0.22^**^	0.07	0.08^*^	0.08	− 0.13^*^	0.09	0.10	0.44^**^	*–*				
10. CTQ_SA	5.33	1.61	0.04	0.06	0.06	− 0.03	− 0.01	0.15^*^	0.15^*^	0.15^*^	0.26^**^	*–*			
11. CTQ_EN	9.59	3.98	0.23^**^	0.16^*^	0.10	0.20^**^	− 0.18^**^	0.23^**^	0.10	0.71^**^	0.36^**^	0.11	*–*		
12. CTQ_PN	6.06	1.79	0.28^**^	0.06^**^	0.04	0.02	− 0.17^**^	0.12	0.09	0.45^**^	0.58^**^	0.21^**^	0.57^**^	*–*	
13. CTQ_TOT	34.95	9.88	0.29^**^	0.18^**^	0.18^**^	0.20^**^	− 0.15^*^	0.24^**^	0.17^**^	0.85^**^	0.67^**^	0.36^**^	0.86^**^	0.74^**^	*–*

Total *N* = 251. *API* appetitive impulsive aggression, *APC* appetitive controlled aggression, *AVI* aversive impulsive aggression, *AVC* aversive controlled aggression.; TRUST, MISTRUST, CREDULITY = Epistemic Trust dimensions; *CTQ_EA* emotional abuse, *CTQ_PA* physical abuse, *CTQ_SA* sexual abuse, *CTQ_EN* emotional neglect, *CTQ_PN* physical neglect, *CTQ_TOT* total score for Childhood Trauma Questionnaire

*p <.05. **p <.01

The four AAS subscales were positively and significantly related to one another, with moderate to large effects. Total CTQ-SF scores were positively related to all AAS subscales, ETMCQ mistrust and credulity subscales, and negatively related to ETMCQ epistemic trust scale, with small effect sizes. All types of aggressive behavior showed positive and significant correlations with Mistrust. Further, API was negatively associated with Trust, while APC and AVI were positively correlated with Credulity.

At the subscale level, Emotional Abuse was positively correlated with both Mistrust and Credulity. Physical Abuse was negatively associated with Trust. Sexual Abuse correlated positively with Mistrust and Credulity. Emotional Neglect was negatively associated with Trust but positively with Mistrust. Lastly, Physical Neglect was negatively correlated with Trust. In relation to aggression, Emotional Abuse was positively correlated with all types of aggressive behavior. Physical Abuse showed a strong correlation with API. Emotional Neglect was positively associated with API, APC, and AVC. Similarly, Physical Neglect was positively correlated with API.

### Cross-Sectional Mediation (“Associational Variable”) Analysis

When considering childhood trauma as a total score (i.e., without differentiating among CTQ-SF subscales), the CTQ-SF maintained a significant direct effect on API, AVI, and AVC, but not APC. Only the indirect effects through Mistrust were significant in explaining the relationship between childhood trauma and all four AAS subscales. Specifically, Mistrust significantly accounted for the relationship between childhood trauma and API (*b* = 0.04, 95% CI [0.001, 0.105]), APC (*b* = 0.06, 95% CI [0.026, 0.101]), AVI (*b* = 0.05, 95% CI [0.022, 0.102]), and AVC (*b* = 0.05, 95% CI [0.012, 0.095]). Table 2 presents the direct effects for all variables, as well as *path a*, *path b*, and direct effects for the mediation analysis. A graphical depiction of the model is reported in Fig. 2 (Model 1).

**Table 2:** Mediation analysis: Childhood trauma total score, epistemic trust, and aggression

Independent Variable	Mediator	Outcome Variable	A	B	C’	Estimate	95% CI	
							LL	UL
CTQ	T	API	−0.15^**^	−0.10	0.25^*^	0.02	−0.003	0.047
CTQ	M	API	0.024^***^	0.17		0.04	**0.001**	**0.105**
CTQ	C	API	0.17^**^	−0.08		−0.01	−0.050	0.014
CTQ	T	APC	−0.15^**^	0.04	0.13	−0.01	−0.030	0.010
CTQ	M	APC	0.24^***^	0.23^***^		0.06	**0.026**	**0.101**
CTQ	C	APC	0.17^**^	0.01		0.00	−0.024	0.032
CTQ	T	AVI	−0.15^**^	0.06	0.13^*^	−0.01	−0.039	0.012
CTQ	M	AVI	0.24^***^	0.22^**^		0.05	**0.022**	**0.102**
CTQ	C	AVI	0.17^**^	0.05		0.01	−0.012	0.041
CTQ	T	AVC	−0.15^**^	0.11	0.18^**^	−0.02	−0.048	0.002
CTQ	M	AVC	0.24^***^	0.19^**^		0.05	**0.012**	**0.095**
CTQ	C	AVC	0.17^**^	−0.02		−0.00	−0.031	0.024

Total *N* = 251. *CTQ* total score for childhood trauma questionnaire, *T* trust, *M* mistrust, *C* credulity (T, M, and C are subscales of the epistemic trust mistrust and credulity questionnaire [ETMCQ]; *API* appetitive impulsive aggression, *APC* appetitive controlled aggression, *AVI* aversive impulsive aggression, *AVC *aversive controlled aggression

A = Path a (effect of CTQ on ETMCQ subscales); B = Path b (effect of ETMCQ subscales on AAS subscales); C’ = Direct effect of CTQ on AAS subscales, controlling for the mediators. The C’ coefficient is only reported in the first row, as it is one coefficient for each. *CI* confidence interval, *LL* lower limit, *UL* upper limit. A graphical depiction of this path analysis is shown in Fig. 2 (Model 1)

*p <.05. **p <.01. ***p<.001

Further analyses explored the role of Trust, Mistrust, and Credulity in accounting for the relation between CTQ-SF and AAS, but examining CTQ-SF subscales as independent variables and AAS subscales as the dependent variables. At this level of analysis, the only direct effects that emerged as significant were between Emotional Abuse and AVI, Emotional Neglect and AVI^1^, as well as Emotional Abuse and AVC. The total indirect effect in the model involving Sexual Abuse, Epistemic Trust subscales, and APC was significant (*b* = 0.03, 95% CI [0.005, 0.070]), as was the total indirect effect predicting AVI (*b* = 0.03, 95% CI [0.008, 0.075]). However, only the specific indirect effects through Mistrust accounting for the relationship between Sexual Abuse and aggressive behavior were significant (see Table 3). Additionally, the total indirect effect in the model involving Emotional Neglect, Epistemic Trust subscales, and API was significant (*b* = 0.057, 95% CI [0.005, 0.140]), but none of the specific indirect effects through individual associational variables were significant. Finally, none of the total nor specific indirect effects involving Emotional Abuse, Physical Abuse, and Physical Neglect as independent variables were significant. A graphical depiction of the model is reported in Fig. 2 (Model 2). The full analysis of results is available in the supplementary material.

**Table 3:** Significant indirect effects: Childhood trauma subscales, epistemic trust subscales, and aggression subscales

Independent Variable	Mediator	Outcome Variable	Estimate	95% CI	
				LL	UL
Sexual Abuse	Mistrust	API	0.03	0.003	0.067
Sexual Abuse	Mistrust	APC	0.03	0.009	0.062
Sexual Abuse	Mistrust	AVI	0.03	0.009	0.060
Sexual Abuse	Mistrust	AVC	0.02	0.005	0.055

Total *N* = 251. *API* appetitive impulsive aggression, *APC* appetitive controlled aggression, *AVI* aversive impulsive aggression, *AVC* aversive controlled aggression

*CI* confidence interval, *LL* lower limit, *UL* upper limit. A graphical depiction of this path analysis is shown in Fig. 2 (Model 2). Full details about this path analysis model are displayed in the Supplementary Material

## Discussion

The present study sought to elucidate associations among different types of childhood traumatic experiences and different types of aggression, further examining whether epistemic trust dimensions may underlie these associations. Inspection of descriptive statistics characterized our sample as presenting low levels of aggression and especially its appetitive forms, as expected for a non-clinical community sample. In line with the Italian validation of the ETMCQ (Liotti et al., 2023), participants had relatively high levels of epistemic trust, and low levels of mistrust and credulity, respectively. Finally, levels of childhood trauma showed minimal representation of sexual abuse (< 10% of the sample), moderate representation of physical abuse and neglect (< 20% of the sample), while almost half of the sample experienced at least mild forms of emotional abuse or neglect, attesting to the widespread prevalence of child maltreatment (WHO, 2006).

Bivariate correlation results showed that, with the exception of sexual abuse, different types of childhood traumatic experiences had moderate to strong relations with one another. This suggests that sexual abuse may involve unique features while the overlap among physical and emotional forms for abuse and neglect is substantial. It should also be noted that, within the CTQ-SF, sexual abuse items are the only ones referring to abuse perpetrated by “someone”, whereas all other subscale items implicitly or explicitly refer to abuse or neglect perpetrated by family members. Epistemic mistrust and credulity were moderately correlated, while trust and mistrust were unrelated and trust and credulity were weakly but positively related. This pattern of associations for the ETMCQ is intermediate between the findings reported in Campbell et al.’s (2021) original validation study – which reported a positive association between mistrust and credulity but a negative association between trust and credulity – and those from Liotti et al.’s (2023) Italian validation study – which reported null relations among the three dimensions of the ETMCQ. Taken together, these findings do suggest that the three dimensions of the ETMCQ capture meaningfully distinct processes within the broader domain of epistemic trust and, specifically, that epistemic trust and mistrust represent orthogonal dimensions rather than two opposites of the same process. Finally, the four types of aggressive behavior were moderately and positively linked to one another, in line with previous findings (e.g., Bant et al., 2025), supporting the idea that the 2-dimensional QVT space represents states rather than traits and that movement is possible across the aggression types.

Looking at bivariate associations across measures of childhood traumatic experiences, epistemic trust dimensions, and aggression, a rather undifferentiated pattern emerged, with some exceptions. First, at the total score level, traumatic childhood experiences were positively related with higher levels of aggression and lower levels of epistemic trust, in line with previous theoretical (Caspi et al., 2014; Fonagy & Allison, 2014; Sprecher et al., 2022; Widom, 1989, 2017) and empirical (Liotti et al., 2023) work. Further, as suggested by indirect evidence (Garofalo & Bogaerts, 2019; Locati et al., 2023; Talia et al., 2021), epistemic trust was associated with aggression, with distinct patterns emerging. Mistrust was the dimension most consistently linked to all forms of aggression. In contrast, low levels of trust were related to thrill-seeking forms of aggression and credulity was related to forms of aggression driven by self-gratification or self-defense. Of note, these associations for trust and credulity had smaller effect sizes than those for mistrust and this pattern of findings was further clarified in the associational variable analyses described below.

Looking at specific types of childhood traumatic experiences, emotional abuse was most consistently linked to lower epistemic trust and higher aggression, followed by emotional neglect. Physical abuse and neglect had relatively less clear and consistent links with childhood traumatic experiences and aggression, although the general pattern was in line with findings obtained at the total score level. Finally, sexual abuse was positively associated with mistrust and credulity but not with any of the other variables. Collectively, these findings suggest a general yet non-specific impact of childhood traumatic experiences on aggression, with emotional forms of maltreatment playing a more significant role. In line with previous studies (Eisenbarth & Garofalo, 2021; McRae et al., 2022) the impact of emotional maltreatment extended to both reactive/impulsive and proactive/controlled forms of aggression. The relevance of emotional abuse and neglect specifically for impairments in epistemic trust makes sense in the context of attachment theory (Fonagy & Allison, 2014, 2023) to the extent that the development of epistemic trust is promoted by a caregiving environment that fosters a feeling of emotional safety in children.

The cross-sectional indirect effects investigated with path analyses helped to shed some light into potential – theory-based – mechanisms that should be explored in longitudinal research as potential mediators. Looking at overall levels of childhood trauma, a clear and consistent pattern emerged, with epistemic mistrust accounting for the effects of childhood traumatic experiences on all four types of aggression. Specifically, epistemic trust fully accounted for the link between childhood traumatic experiences and appetitive-controlled aggression, and partly accounted for links with the other three aggression types. Therefore, when accounting for the overlap – albeit moderate – among epistemic trust, mistrust, and credulity, path analysis results clarified the pattern emerging from the correlation analyses, suggesting that it is the mistrust dimension that is most relevant to an understanding of the mechanisms linking childhood trauma with different manifestations of aggression. These findings are in line with recent reviews (Chokhani et al., 2025) and provide further empirical support for the theoretical argument (Csibra & Gergely, 2009; Garofalo & Bogaerts, 2019; Talia et al., 2021) that experiencing childhood trauma may interfere with the development of epistemic trust, in turn increasing risk for aggressive behavior regardless of whether it is aversively or appetitively driven, and impulsive or controlled. More specifically, our findings suggest that it is not simply a lack of trust, but rather a heightened *mistrust towards others* (i.e. paranoid thinking) that may underlie the childhood trauma-aggression link. Broadly conceived, paranoia encompasses suspicious thoughts and mistrust of others. Howard and Duggan (2022) suggested: “In short, paranoid thinking should be considered an important aspect of antisocial personality, broadly conceived, that in combination with emotional impulsiveness may account for it being associated with violence” (p.146). Believing that others are untrustworthy may therefore be one of the pathways that operates within the so-called cycle of violence (Widom, 2017) and such a tendency could be embedded within broader antisocial personality traits. Clearly, given the cross-sectional design of the study, our findings do not directly support prospective longitudinal relationships over time, but more tentatively yet consistently show that epistemic mistrust explains a significant portion of the variance shared by childhood traumatic experiences and aggression. Conceptually, this suggests that part of the overlap between early traumatic experiences and current aggressive tendencies is explained by individual differences in epistemic trust, and more specifically in epistemic mistrust.

Follow-up exploratory path analysis conducted at the subscale-level to explore direct and indirect effects of childhood traumatic experiences on aggression through epistemic trust provided interesting insights (Humpreys & Zeanah, 2015). In fact, in contrast to correlation analysis, when the overlap among types of trauma was controlled in path analysis, only sexual abuse had consistent indirect effects on all types of aggression through epistemic mistrust, which fully accounted for links between sexual abuse and all four forms of aggression. A significant indirect effect also emerged between emotional neglect and thrill-seeking aggression, but the impact of emotional neglect when controlling for other types of trauma was not consistent and none of the epistemic trust scales significantly explained that association. This pattern of findings conveys two main messages that expand our understanding of the impact of early traumatic experiences. First, it appears to be the shared experience of maltreatment, regardless of the specific type of experience or form of abuse, that drives the development of epistemic trust and aggression. Second, when this shared component is controlled, the specific impact of sexual abuse stands out as more relevant than when shared aspects across types of trauma are not held constant. In other words, all forms of trauma, and perhaps especially emotional abuse and neglect, represent a risk factor for epistemic mistrust and aggression *in general.* However, given *the same level* of traumatic experiences overall, something unique about sexual abuse emerges as a specific risk factor for epistemic mistrust and aggression. In this context, it should be stressed again that the specificity of sexual abuse within the CTQ-SF method of operationalization may also hinge on the fact that, unlike all other CTQ-SF subscales, it enquires about sexual abuse potentially perpetrated by anyone, including people outside the family unit.

### Theoretical and Practical Implications

The findings discussed above underscore the theoretical significance of integrating epistemic trust in developmental models linking childhood trauma and aggression. Specifically, our findings emphasize the importance of longitudinal studies to examine developmental processes linking experiences of child maltreatment with impairments in epistemic trust and later aggression. In this context, we argue that more attention should be paid to shared mechanisms that link different forms of maltreatment with different forms of aggression. While it is tempting to search for links between specific forms of maltreatment and specific forms of aggression, this might lead to an artificial fragmentation of the literature on the topic. Further, our findings emphasize the theoretical importance of distinguishing between the absence of a protective factor (in this case, epistemic trust) and the presence of a risk factor (in this case, epistemic mistrust) for the development and manifestation of maladaptive behaviors such as aggression. When examined together, it appears that it is the presence of genuinely maladaptive forms of epistemic mistrust, rather than the absence of adaptive forms of epistemic trust, that is robustly associated with aggressive behavior. Conceptually, a recent review (Chokhani et al., 2025) has suggested that trust can play a role in explaining links between early adversity and later maladjustment by contributing to increased social thinning (defined as a reduction in the quantity and quality of interpersonal relationships and the subjective experience of loneliness) and stress generation (defined as an individual liability to experience interpersonal stressors and ruptures; McCrory et al., 2022). While these mechanisms are typically implied in explosive and defensive forms of aggression, our analysis guided by the QVT model also elucidated ways in which our understanding of aggression can be furthered by moving beyond a rigid dichotomy between reactive and proactive forms of aggression wherein the latter is often considered less impacted by environmental and individual differences aspects.

Practical implications should be interpreted cautiously due to the non-clinical nature of our sample. Nevertheless, we see no conceptual or empirical reason to expect different patterns in populations with higher levels of psychological maladjustment. The findings underscore the value of early interventions that address not only general trauma-related distress but also the development of epistemic trust. Clinically, this highlights the importance of fostering secure, emotionally validating relationships as a means of mitigating aggressive tendencies in individuals with childhood trauma histories.

Prevention policies and practices may likewise benefit from emphasizing the potentially far-reaching consequences of early traumatic experiences—even when, as in our community sample, these experiences occur at relatively low levels. When prevention efforts cannot be deployed before trauma exposure, efforts of secondary prevention should focus on flexibly promoting trust in others and preventing rigid, globalized tendencies to either trust or mistrust everyone. This aligns with accounts suggesting that adaptive social functioning depends on calibrating trust to contextual demands (Schilke et al., 2021). At a policy level, our results support recent proposals to embed understandings of trust—along with related constructs such as mentalizing and agency—within broader social systems, including schools, social care, and healthcare services that often serve as first points of contact for trauma-exposed children and youth (Chokhani et al., 2025). Finally, our findings extend existing knowledge of the environmental and individual-difference factors associated with all forms of aggression, including those typically regarded as predatory. This understanding should not imply exculpation but rather call for greater societal attention to the factors that contribute to aggressive behavior of all kinds (Fonagy, 2003).

### Limitations

Findings of this study should be interpreted in light of its limitations. The first and obvious limitation pertains to its mono-method and mono-sample design that relied exclusively on a convenience sample drawn from the general population, and on self-report measures of the key constructs. Specifically, although we strove to maximize the representativeness of our sample in terms of its socio-demographic characteristics, the generalizability of our findings is limited by non-inclusion of minority groups. Second, cross-sectional mediation analyses cannot directly test the implied temporal effects assumed in the theoretical mediation model (O’Laughlin et al., 2018; Maxwell & Cole, 2007). However, our ordering of key variables in the path analyses was based on the theoretical grounds articulated in the introduction, supporting the use of cross-sectional mediation analysis as a first step to stimulate further investigation in longitudinal studies (Rohrer et al., 2022; Winer et al., 2016). Clearly, our findings by no means support temporal, let alone causal, claims regarding the relationships investigated. However, they may represent a first step of “associational variable analysis” (Weems, 2025) identifying potential mediators and intermediary variables. In our study, epistemic trust may be an associational variable that links childhood trauma and aggression, being correlated with both. Further, epistemic trust partly accounts for the association between childhood trauma and aggression providing a correlational link between two. As such, it holds promise to be later scrutinized as mediators with appropriate longitudinal designs. Relatedly, we specified a fully saturated model, which cannot be used to assess model fit or misspecification.Third, while we tested a novel potential variable accounting for widely established link between childhood trauma and aggression, our study design did not allow us to test head-to-head comparisons between epistemic trust and other potential mediators that have emerged from previous research. Notwithstanding, we do hope these limitations may be overcome in future studies leveraging longitudinal study designs that rely on multi-method assessment to test prospective associations between childhood trauma and aggression and pit against one another several potential mediators underlying these associations.

## Conclusions

These limitations notwithstanding, the present study replicates and extends current knowledge on the so-called cycle of violence by suggesting that (1) childhood traumatic experiences are robust yet non-specific risk factors that can compromise the development of epistemic trust and increase aggressive behavior; (2) a tendency to consider others as untrustworthy sources of information may be specifically linked to increased aggression manifesting in different forms and functions, and may explain the childhood trauma-aggression link; and (3) it is likely that rather than specific types of trauma, it is something shared in the experience of different forms of child maltreatment that confers the greatest risk for epistemic mistrust and aggression, with sexual abuse emerging as a specific risk factor given same levels of other traumatic experiences. While preliminary, these findings suggest that epistemic trust may be a critical construct that merits further consideration in current theories and in prevention and treatment efforts aimed at mitigating the negative consequences of child abuse and neglect.

## Supplementary Information

Below is the link to the electronic supplementary material.

Supplementary Material 1 (DOCX 36.4 KB)

## Data Availability

Data can be obtained from the corresponding author upon request.

## Footnotes

1 The direct effect between Emotional Neglect and AVI was negative in sign, suggesting a potential statistical suppression effect.

## References

[CR1] Badoud, D., Luyten, P., Fonseca-Pedrero, E., Eliez, S., Fonagy, P., & Debbané, M. (2015). The French version of the Reflective Functioning Questionnaire: Validity data for Adolescents and adults and its association with non-suicidal self-injury. *PLoS One,**10*(12), Article e0145892. 10.1371/journal.pone.0145892 26714319 PMC4694697

[CR2] Baldwin, J. R., Wang, B., Karwatowska, L., Schoeler, T., Tsaligopoulou, A., Munafò, M. R., & Pingault, J. B. (2023). Childhood maltreatment and mental health problems: A systematic review and meta-analysis of quasi-experimental studies. *American Journal of Psychiatry,**180*(2), 117–126. 10.1176/appi.ajp.20220174 36628513 PMC7614155

[CR3] Bant,L., Bogaerts, S., Howard, R., Mazzeschi, C., & Garofalo, C. (2025). The quadripartite violence typology: Further validation of the Angry Aggression Scale in a Dutch community sample. *Psychology of Violence, 15*(6), 712–729. 10.1037/vio0000594

[CR4] Bernard, K., Frost, A., Bennett, C. B., & Lindhiem, O. (2017). Maltreatment and diurnal cortisol regulation: A meta-analysis. *Psychoneuroendocrinology,**78*, 57–67. 28167370 10.1016/j.psyneuen.2017.01.005

[CR5] Bernstein, D. P., Fink, L., Handelsman, L., & Foote, J. (1998). Childhood trauma questionnaire. *Assessment of family violence: A handbook for researchers and practitioners.*

[CR6] Bernstein, D. P., Stein, J. A., Newcomb, M. D., Walker, E., Pogge, D., Ahluvalia, T., Stokes, J., Handelsman, L., Medrano, M., Desmond, D., & Zule, W. (2003). Development and validation of a brief screening version of the Childhood Trauma Questionnaire. *Child Abuse & Neglect,**27*(2), 169–190. 10.1016/s0145-2134(02)00541-0 12615092

[CR7] Bjørnebekk, G., & Howard, R. (2012). Validation of a motivation-based typology of angry aggression among antisocial youths in Norway. *Behavioral Sciences & the Law*, *30*(2), 167–180. 10.1002/bsl.2007 22388964

[CR8] Campbell, C., Tanzer, M., Saunders, R., Booker, T., Allison, E., Li, E., O’Dowda, C., Luyten, P., & Fonagy, P. (2021). Development and validation of a self-report measure of epistemic trust. *PLoS One,**16*(4), Article e0250264. 10.1371/journal.pone.0250264 33861805 PMC8051785

[CR9] Caspi, A., Houts, R. M., Belsky, D. W., Goldman-Mellor, S. J., Harrington, H., Israel, S., Meier, M. H., Ramrakha, S., Shalev, I., Poulton, R., & Moffitt, T. E. (2014). The p factor: One general psychopathology factor in the structure of psychiatric disorders? *Clinical Psychological Science,**2*(2), 119–137. 10.1177/2167702613497473 25360393 PMC4209412

[CR10] Chokhani, R., Lloyd, A., Viding, E., Gerin, M., & McCrory, E. (2025). Linking interpersonal childhood adversity to mental health: A scoping review of trust, mentalizing, agency and interpersonal emotion regulation as candidate social-transactional mechanisms. *Clinical Psychology Review.* Manuscript accepted for publication. 10.1016/j.cpr.2026.102722 41762964

[CR11] Christian, E., & Sellbom, M. (2016). Development and validation of an expanded version of the Three-Factor Levenson Self-Report psychopathy scale. *Journal of Personality Assessment*, *98*(2), 155–168. 10.1080/00223891.2015.1068176 26542172

[CR12] Csibra, G., & Gergely, G. (2009). Natural pedagogy. *Trends in Cognitive Sciences*, *13*(4), 148–153. 10.1016/j.tics.2009.01.005 19285912

[CR13] DeLisi, M., Fox, B. H., Fully, M., & Vaughn, M. G. (2018). The effects of temperament, psychopathy, and childhood trauma among delinquent youth: A test of DeLisi and Vaughn’s temperament-based theory of crime. *International Journal of Law and Psychiatry,**57*, 53–60. 10.1016/j.ijlp.2018.01.006 29548504

[CR14] Dodge, K. A., Pettit, G. S., Bates, J. E., & Valente, E. (1995). Social information-processing patterns partially mediate the effect of early physical abuse on later conduct problems. *Journal of Abnormal Psychology,**104*(4), 632–643. 10.1037/0021-843x.104.4.632 8530766

[CR15] Dutton, D. G. (2007). *The abusive personality: Violence and control in intimate relationships* (2nd ed.). The Guilford Press.

[CR16] Ehrensaft, M. K., Cohen, P., Brown, J., Smailes, E., Chen, H., & Johnson, J. G. (2003). Intergenerational transmission of partner violence: A 20-year prospective study. *Journal of Consulting and Clinical Psychology,**71*(4), 741–753. 10.1037/0022-006x.71.4.741 12924679

[CR17] Eisenbarth, H., & Garofalo, C. (2021). The role of psychopathic traits in explaining associations between childhood traumatic experiences and aggression. *Journal of Personality Disorders,**35*(Supple C), 38–55. 10.1521/pedi_2021_35_507 33779279

[CR18] Fitton, L., Yu, R., & Fazel, S. (2020). Childhood maltreatment and violent outcomes: A systematic review and meta-analysis of prospective studies. *Trauma, Violence & Abuse,**21*(4), 754–768. 10.1177/1524838018795269 30122119

[CR19] Fonagy, P. (2003). Towards a developmental understanding of violence. *British Journal of Psychiatry,**183*(3), 190–192. 10.1192/bjp.183.3.190 12948988

[CR20] Fonagy, P., & Allison, E. (2014). The role of mentalizing and epistemic trust in the therapeutic relationship. *Psychotherapy*, *51*(3), 372–380. 10.1037/a0036505 24773092

[CR21] Fonagy, P., & Allison, E. (2023). Beyond mentalizing: Epistemic trust and the transmission of culture. *The Psychoanalytic Quarterly,**92*(4), 599–640. 10.1080/00332828.2023.2290023 38095858

[CR22] Fonagy, P., Luyten, P., & Allison, E. (2015). Epistemic petrification and the restoration of epistemic trust: A new conceptualization of borderline personality disorder and its psychosocial treatment. *Journal of Personality Disorders,**29*(5), 575–609. 10.1521/pedi.2015.29.5.575 26393477

[CR23] Fonagy, P., Luyten, P., Allison, E., & Campbell, C. (2017). What we have changed our minds about: Part 2. Borderline personality disorder, epistemic trust and the developmental significance of social communication. *Borderline Personality Disorder and Emotion Dysregulation,**4*, Article 9. 10.1186/s40479-017-0062-8 28405338 PMC5387344

[CR24] Garofalo, C., & Bogaerts, S. (2019). Attachment and personality disorders among child molesters: The role of trust. *Sexual Abuse*, *31*(1), 97–124. 10.1177/1079063217720928 28735566 PMC6330767

[CR25] Grassi-Oliveira, R., & Stein, L. M. (2008). Childhood maltreatment associated with PTSD and emotional distress in low-income adults: The burden of neglect. *Child Abuse & Neglect*, *32*(12), 1089–1094. 10.1016/j.chiabu.2008.05.008 19036445

[CR26] Howard, R. C. (2009). The neurobiology of affective dyscontrol: Implications for understanding ‘dangerous and severe personality disorder.’ In R. C. Howard & M. McMurran (Eds.), *Personality, personality disorder and violence* (pp. 133–174). J. Wiley & Sons.

[CR27] Howard, R. C., & Duggan, C. (2022). *Antisocial Personality. Theory, Research, treatment*. Cambridge University Press.

[CR28] Howard, R. C., Howells, K., Jinks, M., & McMurran, M. (2009). A quadripartite typology of violence (QTV): Relationships with functions of aggression in violent youths. *Violence in Clinical Psychiatry the Netherlands: Kavanah*, 342–345.

[CR29] Humphreys, K. L., & Zeanah, C. H. (2015). Deviations from the expectable environment in early childhood and emerging psychopathology. *Neuropsychopharmacology,**40*(1), 154–170. 10.1038/npp.2014.165 24998622 PMC4262894

[CR30] Humphreys, K. L., LeMoult, J., Wear, J. G., Piersiak, H. A., Lee, A., & Gotlib, I. H. (2020). Child maltreatment and depression: A meta-analysis of studies using the Childhood Trauma Questionnaire. *Child Abuse & Neglect,**102*, Article 104361. 10.1016/j.chiabu.2020.104361 32062423 PMC7081433

[CR31] IBM Corp. (2020). *IBM SPSS statistics for Windows, version 27.0*. IBM Corp.

[CR32] Kim, J., & Cicchetti, D. (2006). Longitudinal trajectories of self-system processes and depressive symptoms among maltreated and nonmaltreated children. *Child Development,**77*(3), 624–639. 10.1111/j.1467-8624.2006.00894.x 16686792 PMC1551975

[CR33] LaMotte, A. D., Taft, C. T., & Weatherill, R. P. (2016). Mistrust of others as a mediator of the relationship between trauma exposure and use of partner aggression. *Psychological Trauma: Theory, Research, Practice, and Policy,**8*(4), 535–540. 10.1037/tra0000157 27348070 PMC4924534

[CR34] Leenarts, L. E., Diehle, J., Doreleijers, T. A., Jansma, E. P., & Lindauer, R. J. (2013). Evidence-based treatments for children with trauma-related psychopathology as a result of childhood maltreatment: A systematic review. *European Child & Adolescent Psychiatry,**22*(5), 269–283. 10.1007/s00787-012-0367-5 23266844

[CR35] Liotti, M., Milesi, A., Spitoni, G. F., Tanzilli, A., Speranza, A. M., Parolin, L., Campbell, C., Fonagy, P., Lingiardi, V., & Giovanardi, G. (2023). Unpacking trust: The Italian validation of the Epistemic Trust, Mistrust, and Credulity Questionnaire (ETMCQ). *PLoS One,**18*(1), Article e0280328. 10.1371/journal.pone.0280328 36701301 PMC9879475

[CR36] Liu, R. T. (2017). Childhood adversities and depression in adulthood: Current findings and future directions. *Clinical Psychology Science and Practice,**24*(2), 140–153. 10.1111/cpsp.12190 28924333 PMC5600284

[CR37] Locati, F., Benzi, I. M. A., Milesi, A., Campbell, C., Midgley, N., Fonagy, P., & Parolin, L. (2023). Associations of mentalization and epistemic trust with internalizing and externalizing problems in adolescence: A gender-sensitive structural equation modeling approach. *Journal of Adolescence,**95*(8), 1564–1577. 10.1002/jad.12226 37500187

[CR38] Luyten, P., Campbell, C., Allison, E., & Fonagy, P. (2020). The mentalizing approach to psychopathology: State of the art and future directions. *Annual Review of Clinical Psychology,**16*, 297–325. 10.1146/annurev-clinpsy-071919-015355 32023093

[CR39] Main, M., & Cassidy, J. (1988). Categories of response to reunion with the parent at age 6: Predictable from infant attachment classifications and stable over a 1-month period. *Developmental Psychology*, *24*(3), 415–426. 10.1037/0012-1649.24.3.415

[CR40] Malcorps, S., Vliegen, N., & Luyten, P. (2024). Childhood adversity and adolescent acting-out behaviors: The mediating role of mentalizing difficulties and epistemic vigilance. *European Child & Adolescent Psychiatry*, *33*(7), 2153–2162. 10.1007/s00787-023-02302-9 37787820

[CR41] Maxfield, M. G., & Widom, C. S. (1996). The cycle of violence. Revisited 6 years later. *Archives of Pediatrics & Adolescent Medicine,**150*(4), 390–395. 10.1001/archpedi.1996.02170290056009 8634734

[CR42] Maxwell, S. E., & Cole, D. A. (2007). Bias in cross-sectional analyses of longitudinal mediation. *Psychological Methods,**12*(1), 23–44. 10.1037/1082-989X.12.1.23 17402810

[CR43] McCrory, E., Foulkes, L., & Viding, E. (2022). Social thinning and stress generation after childhood maltreatment: A neurocognitive social transactional model of psychiatric vulnerability. *The Lancet Psychiatry*, *9*(10), 828–837. 10.1016/S2215-0366(22)00202-4 35926524

[CR44] McRae, E. M., Stoppelbein, L., O’Kelley, S. E., Fite, P. K., & Smith, S. B. (2022). Pathways from child maltreatment to proactive and reactive aggression: The role of posttraumatic stress symptom clusters. *Psychological Trauma: Theory, Research, Practice, and Policy,**14*(3), 357–366. 10.1037/tra0001051 34516224

[CR45] Meddeb, A., Garofalo, C., Karlén, M. H., & Wallinius, M. (2023). Emotion dysregulation–a bridge between ACE and aggressive antisocial behavior. *Journal of Criminal Justice,**88*, Article 102110.

[CR46] Meinck, F., Lu, M., Suresh, D., Cetin, M., Neelakantan, L., Hemady, C., Franchino-Olsen, H., Woollett, N., Melendez-Torres, G., Gonzalez, A., & Christofides, N. (2025). What are the mechanisms underpinning intergenerational transmission of violence perpetration? A realist review. *Trauma Violence & Abuse*. 10.1177/15248380251361468. Advance online publication. PMC13578666 40888527

[CR47] Muthén, L. K., & Muthén, B. (1998–2017). Mplus user’s guide. Muthén & Muthén.

[CR48] Norman, R. E., Byambaa, M., De, R., Butchart, A., Scott, J., & Vos, T. (2012). The long-term health consequences of child physical abuse, emotional abuse, and neglect: A systematic review and meta-analysis. *PLoS Medicine,**9*(11), Article e1001349. 10.1371/journal.pmed.1001349 23209385 PMC3507962

[CR49] O’Laughlin, K. D., Martin, M. J., & Ferrer, E. (2018). Cross-sectional analysis of longitudinal mediation processes. *Multivariate Behavioral Research,**53*(3), 375–402. 10.1080/00273171.2018.1454822 29624079

[CR50] Ogloff, J. R., Cutajar, M. C., Mann, E., Mullen, P., Wei, F. T. Y., Hassan, H. A. B., & Yih, T. H. (2012). Child sexual abuse and subsequent offending and victimisation: A 45 year follow-up study. *Trends and Issues in Crime and Criminal Justice,**440*, 1–6.

[CR51] Perry, B. D. (2004). *Maltreatment and the developing child: How early childhood experience shapes child and culture*. The Margaret Mc Cain Lecture Series.

[CR52] Petersen, A. C., Joseph, J., & Feit, M. (2014). Committee on child maltreatment Research, Policy, and practice for the next decade: Phase II, board on children, Youth, and Families, committee on law and Justice, Institute of medicine. In N. R. Council (Ed.), *New directions in child abuse and neglect research*. National Academies Press (US). 24757747

[CR53] Rohrer, J. M., Hünermund, P., Arslan, R. C., & Elson, M. (2022). That’s a lot to process! Pitfalls of popular path models. *Advances in Methods and Practices in Psychological Science*. 10.1177/25152459221095827

[CR54] Sacchi, C., Vieno, A., & Simonelli, A. (2018). Italian validation of the childhood trauma questionnaire-short form on a college group. *Psychological Trauma: Theory, Research, Practice and Policy,**10*(5), 563–571. 10.1037/tra0000333 29016156

[CR55] Schilke, O., Reimann, M., & Cook, K. S. (2021). Trust in social relations. *Annual Review of Sociology,**47*(1), 239–259. 10.1146/annurev-soc-082120-082850

[CR56] Schmitz, S. E., Niedtfeld, I., Lane, S. P., Seitz, K. I., & Hepp, J. (2023). Negative affect provides a context for increased distrust in the daily lives of individuals with a history of childhood maltreatment. *Journal of Traumatic Stress,**36*, 808–819. 10.1002/jts.22951 37437133

[CR57] Schuck, A. M., & Widom, C. S. (2021). The roles of housing, financial, and food insecurities in understanding the relationship between childhood neglect and violence in adulthood. *PLoS One,**16*(3), Article e0246682. 10.1371/journal.pone.0246682 33657121 PMC7928485

[CR58] Schwarzer, N. H., Nolte, T., Fonagy, P., & Gingelmaier, S. (2021). Mentalizing mediates the association between emotional abuse in childhood and potential for aggression in non-clinical adults. *Child Abuse & Neglect,**115*, Article 105018. 10.1016/j.chiabu.2021.105018 33676103

[CR59] Shields, A., & Cicchetti, D. (1998). Reactive aggression among maltreated children: The contributions of attention and emotion dysregulation. *Journal of Clinical Child Psychology*, *27*(4), 381–395. 10.1207/s15374424jccp2704_2 9866075

[CR60] Sperber, D., Clément, F., Heintz, C., Mascaro, O., Mercier, H., Origgi, G., & Wilson, D. (2010). Epistemic vigilance. *Mind & Language*, *25*(4), 359–393. 10.1111/j.1468-0017.2010.01394.x

[CR61] Sprecher, E. A., Li, E., Sleed, M., & Midgley, N. (2022). ‘Trust me, we can sort this out’: A theory-testing case study of the role of epistemic trust in fostering relationships. *Qualitative Research in Psychology,**19*(4), 1117–1142. 10.1080/14780887.2022.2033898

[CR62] Stagaki, M., Nolte, T., Feigenbaum, J., King-Casas, B., Lohrenz, T., Fonagy, P., Personality and Mood Disorder Research Consortium, & Montague, P. R. (2022). The mediating role of attachment and mentalising in the relationship between childhood maltreatment, self-harm and suicidality. *Child Abuse & Neglect,**128*, Article 105576. 10.1016/j.chiabu.2022.105576 35313127 PMC10466023

[CR63] Suchman, N. E., DeCoste, C., Borelli, J. L., & McMahon, T. J. (2018). Does improvement in maternal attachment representations predict greater maternal sensitivity, child attachment security and lower rates of relapse to substance use? A second test of Mothering from the Inside Out treatment mechanisms. *Journal of Substance Abuse Treatment,**85*, 21–30. 10.1016/j.jsat.2017.11.006 29291768 PMC5753433

[CR64] Talia, A., Duschinsky, R., Mazzarella, D., Hauschild, S., & Taubner, S. (2021). Epistemic trust and the emergence of conduct problems: Aggression in the service of communication. *Frontiers in Psychiatry,**12*, Article 710011. 10.3389/fpsyt.2021.710011 34630177 PMC8494977

[CR65] Taubner, S., Zimmermann, L., Ramberg, A., & Schröder, P. (2016). Mentalization mediates the relationship between early maltreatment and potential for violence in adolescence. *Psychopathology*, *49*(4), 236–246. 10.1159/000448053 27548462

[CR66] Tironi, M., Charpentier Mora, S., Liotti, M., Fiorini Bincoletto, A., Tanzilli, A., Cavanna, D., Lingiardi, V., Speranza, A. M., Giovanardi, G., & Bizzi, F. (2024). Adverse childhood experiences and psychological maladjustment in adolescence: The protective role of epistemic trust, mentalized affectivity, and reflective functioning. *Journal of Clinical Psychology*. 10.1002/jclp.23733 39101491

[CR67] Weeland, J., Overbeek, G., Castro, B. O., & Matthys, W. (2015). Underlying mechanisms of gene–environment interactions in externalizing behavior: A systematic review and search for theoretical mechanisms. *Clinical Child and Family Psychology Review*, *18*(4), 413–442. 10.1007/s10567-015-0196-4 26537239 PMC4637001

[CR68] Weems, C. F. (2025). An alternative framework for nonexperimental cross-sectional mediation studies: Associational variable analysis. *Psychological Methods*. Advance online publication. 10.1037/met0000774 40608441

[CR69] Widom,C. S. (1989). The cycle of violence. *Science, 244*(4901), 160–166. 10.1126/science.2704995 2704995

[CR70] Widom, C. S. (2017). Long‐term impact of childhood abuse and neglect on crime and violence. Clinical Psychology: Science and Practice, 24(2), 186–202. 10.1037/h0101743

[CR71] Winer,E. S., Cervone, D., Bryant, J., McKinney, C., Liu, R. T., & Nadorff, M. R. (2016). Distinguishing mediational models and analyses in clinical psychology: Atemporal associations do not imply causation. *Journal of Clinical Psychology, 72*(9), 947–955. 10.1002/jclp.22298 27038095

[CR72] World Health Organization and International Society for Prevention of Child Abuse and Neglect. (2006). *Preventing child maltreatment: A guide to taking action and generating evidence*. WHO Press.

