# Supplementary material for: Childhood Trauma and Aggression: The Role of Epistemic Trust
Source: J Child Adolesc Trauma. 2026 Mar 17;19(3):1581–95. doi: 10.1007/s40653-026-00841-2 (PMC13546256; doi:10.1007/s40653-026-00841-2)
Supplement: Supplementary file 1 [file 40653_2026_841_MOESM1_ESM.docx]

*Childhood Trauma and Aggression: The Role of Epistemic Trust*

**Supplementary Material**

**Full mediation analysis results**

| Indipendent Variable | Mediator | Outcome Variable | A | B | C' | Estimate | 95% CI | |
| --- | --- | --- | --- | --- | --- | --- | --- | --- |
|  |  |  |  |  |  |  | *LL* | *UL* |
| EA | T | API | **0.194^*^** | -0.097 | 0.127 | -0.019 | -0.075 | 0.004 |
| EA | M | API | 0.141 | **0.193^*^** |  | 0.027 | -0.002 | .004 |
| EA | C | API | **0.210^*^** | -0.079 |  | -0.016 | -0.082 | 0.012 |
| PA | T | API | -0.092 | -0.097 | 0.064 | 0.009 | -0.006 | 0.048 |
| PA | M | API | -0.039 | **0.193^*^** |  | -0.007 | -0.045 | 0.013 |
| PA | C | API | -0.012 | -0.079 |  | 0.001 | -0.013 | 0.028 |
| SA | T | API | 0.023 | -0.097 | -0.050 | -0.002 | -0.022 | 0.005 |
| SA | M | API | **0.132^**^** | **0.193^*^** |  | 0.025 | **0.003** | **0.067** |
| SA | C | API | **0.125^*^** | -0.079 |  | -0.010 | -0.042 | 0.009 |
| EN | T | API | **-0.248^*^** | -0.097 | -0.030 | 0.024 | -0.005 | 0.083 |
| EN | M | API | 0.146 | **0.193^*^** |  | 0.028 | -0.002 | 0.105 |
| EN | C | API | -0.068 | -0.079 |  | 0.005 | -0.007 | 0.050 |
| PN | T | API | -0.063 | -0.097 | 0.183 | 0.006 | -0.008 | 0.045 |
| PN | M | API | -0.032 | **0.193^*^** |  | -0.006 | -0.048 | 0.013 |
| PN | C | API | 0.019 | -0.079 |  | -0.001 | -0.037 | 0.013 |
| EA | T | APC | **0.194^*^** | 0.029 | 0.166 | 0.006 | -0.016 | 0.040 |
| EA | M | APC | 0.141 | **0.226^***^** |  | 0.032 | -0.005 | 0.090 |
| EA | C | APC | **0.210^*^** | 0.011 |  | 0.002 | -0.033 | 0.043 |
| PA | T | APC | -0.092 | 0.029 | 0.005 | -0.003 | -0.030 | 0.007 |
| Indipendent Variable | Mediator | Outcome Variable | A | B | C' | Estimate | 95% CI | |
|  |  |  |  |  |  |  | *LL* | *UL* |
| PA | M | APC | -0.039 | **0.226^***^** |  | -0.009 | -0.039 | 0.021 |
| PA | C | APC | -0.012 | 0.011 |  | 0.000 | -0.017 | 0.012 |
| SA | T | APC | 0.023 | 0.029 | 0.004 | 0.001 | -0.004 | 0.013 |
| SA | M | APC | **0.132^**^** | **0.226^***^** |  | 0.030 | **0.009** | **0.062** |
| SA | C | APC | **0.125^*^** | 0.011 |  | 0.001 | -0.020 | 0.025 |
| EN | T | APC | **-0.248^*^** | 0.029 | 0.025 | -0.007 | -0.046 | 0.021 |
| EN | M | APC | 0.146 | **0.226^***^** |  | 0.033 | -0.007 | 0.091 |
| EN | C | APC | -0.068 | 0.011 |  | -0.001 | -0.028 | 0.014 |
| PN | T | APC | -0.063 | 0.029 | -0.057 | -0.002 | -0.026 | 0.007 |
| PN | M | APC | -0.032 | **0.226^***^** |  | -0.007 | -0.041 | 0.022 |
| PN | C | APC | 0.019 | 0.011 |  | 0.000 | -0.015 | 0.019 |
| EA | T | AVI | **0.194^*^** | 0.023 | **0.393^***^** | 0.004 | -0.022 | 0.047 |
| EA | M | AVI | 0.141 | **0.220^***^** |  | 0.031 | -0.003 | 0.092 |
| EA | C | AVI | **0.210^*^** | 0.031 |  | 0.006 | -0.017 | 0.046 |
| PA | T | AVI | -0.092 | 0.023 | -0.031 | -0.002 | -0.035 | 0.011 |
| PA | M | AVI | -0.039 | **0.220^***^** |  | -0.008 | -0.039 | 0.021 |
| PA | C | AVI | -0.012 | 0.031 |  | 0.000 | -0.019 | 0.010 |
| SA | T | AVI | 0.023 | 0.023 | 0.002 | 0.001 | -0.005 | 0.015 |
| SA | M | AVI | **0.132^**^** | **0.220^***^** |  | 0.029 | **0.009** | **0.060** |
| SA | C | AVI | **0.125^*^** | 0.031 |  | 0.004 | -0.011 | 0.028 |
| EN | T | AVI | **-0.248^*^** | 0.023 | **-0.201^*^** | -0.006 | -0.051 | 0.032 |
| EN | M | AVI | 0.146 | **0.220^***^** |  | 0.032 | -0.006 | 0.096 |
| EN | C | AVI | -0.068 | 0.031 |  | -0.002 | -0.030 | 0.008 |
| Indipendent Variable | Mediator | Outcome Variable | A | B | C' | Estimate | 95% CI | |
|  |  |  |  |  |  |  | *LL* | *UL* |
| PN | T | AVI | -0.063 | 0.023 | -0.027 | -0.001 | -0.031 | 0.011 |
| PN | M | AVI | -0.032 | **0.220^***^** |  | -0.007 | -0.045 | 0.019 |
| PN | C | AVI | 0.019 | 0.031 |  | 0.001 | -0.010 | 0.024 |
| EA | T | AVC | **0.194^*^** | 0.082 | **0.284^**^** | 0.016 | -0.005 | 0.071 |
| EA | M | AVC | 0.141 | **0.175^*^** |  | 0.025 | -0.002 | 0.083 |
| EA | C | AVC | **0.210^*^** | -0.016 |  | -0.003 | -0.048 | 0.026 |
| PA | T | AVC | -0.092 | 0.082 | 0.030 | -0.008 | -0.048 | 0.005 |
| PA | M | AVC | -0.039 | **0.175^*^** |  | -0.007 | -0.035 | 0.014 |
| PA | C | AVC | -0.012 | -0.016 |  | 0.000 | -0.010 | 0.017 |
| SA | T | AVC | 0.023 | 0.082 | -0.072 | 0.002 | -0.005 | 0.020 |
| SA | M | AVC | **0.132^**^** | **0.175^*^** |  | 0.023 | **0.005** | **0.055** |
| SA | C | AVC | **0.125^*^** | -0.016 |  | -0.002 | -0.027 | 0.017 |
| EN | T | AVC | **-0.248^*^** | 0.082 | 0.052 | -0.020 | -0.083 | 0.006 |
| EN | M | AVC | 0.146 | **0.175^*^** |  | 0.026 | -0.003 | 0.086 |
| EN | C | AVC | -0.068 | -0.016 |  | 0.001 | -0.011 | 0.031 |
| PN | T | AVC | -0.063 | 0.082 | -0.149 | -0.005 | -0.042 | 0.007 |
| PN | M | AVC | -0.032 | **0.175^*^** |  | -0.006 | -0.040 | 0.015 |
| PN | C | AVC | 0.019 | -0.016 |  | 0.000 | -0.022 | 0.012 |

*Note.* Total *N* = 251. EA = Emotional Abuse; PA = Physical Abuse; SA = Sexual Abuse; EN = Emotional Neglect; PN = Physical Neglect; T = Trust; M = Mistrust; C = Credulity; API = Appetitive impulsive Aggression; APC = Appetitive Controlled Aggression; AVI = Aversive Impulsive Aggression; AVC = Aversive Controlled Aggression. A = Path a (effect of CTQ subscales on ET subscales); B = Path b (effect of ET subscales on AAS subscales); C’ = Direct effect of CTQ subscales on AAS subscales, controlling for the mediators. The C’ coefficient is only reported in the first row, as it is one coefficient for each model. CI = confidence interval; *LL* = lower limit; *UL* = upper limit. See Figure 2 (Model 2) for a graphical depiction of the path analysis.

**p* < .05. ***p* < .01. ****p* < .001
